# The effectiveness of transcranial magnetic stimulation for dysphagia in stroke patients: an umbrella review of systematic reviews and meta-analyses

**DOI:** 10.3389/fnhum.2024.1355407

**Published:** 2024-03-14

**Authors:** Anastasios M. Georgiou, Phivos Phylactou, Maria Kambanaros

**Affiliations:** ^1^The Cyprus Rehabilitating Aphasia and Dysphagia (C-RAD) Lab, Department of Rehabilitation Sciences, Cyprus University of Technology, Limassol, Cyprus; ^2^The Brain and Neurorehabilitation Lab, Department of Rehabilitation Sciences, Cyprus University of Technology, Limassol, Cyprus; ^3^School of Physical Therapy, University of Western Ontario, London, ON, Canada; ^4^The Gray Centre of Mobility and Activity, Parkwood Institute, London, ON, Canada

**Keywords:** dysphagia, brain stimulation, TMS, umbrella review, meta-analysis, scientific rigor

## Abstract

Numerous studies have explored the use of repetitive Transcranial Magnetic Stimulation (rTMS) intervention in post-stroke dysphagia. The primary aim of this umbrella review was to appraise the methodological quality of systematic reviews (SRs), with and without meta-analyses (MAs), that synthesized the findings of randomized controlled trials (RCTs) exploring the effectiveness of rTMS in the management of dysphagia post-stroke. A secondary aim of was to evaluate the consistency and reliability of translational implications of rTMS for swallowing recovery after stroke across these SRs and MAs. We searched several databases from inception to the 14th of May 2023, to identify SRs and MAs that examined the effectiveness of rTMS in the management of dysphagia post-stroke. The methodological quality of the included studies was evaluated utilizing the AMSTAR 2 (A Measurement Tool to Assess Systematic Reviews) instrument. To investigate the extent of literature overlap among the primary studies included in the SRs, the Graphical Overview of Evidence (GROOVE) was utilized. Of the 19 SRs that were identified, two studies received low quality ratings, while the rest (17) were rated with critically low quality based on the AMSTAR 2 rating. A high literature overlap across the SRs was observed. In all SRs and MAs reviewed, there was a consistent presence of at least some significant evidence supporting the effectiveness of rTMS in enhancing swallowing outcomes for individuals with dysphagia post-stroke, that is, all MAs reported at least a moderate overall effect in favor of rTMS (SMD range = [0.59, 6.23]). While rTMS shows promise for improving dysphagia post-stroke, the current evidence remains limited and inconclusive due to the methodological flaws observed in the published SRs and their respective MAs on the topic so far. Concerning the limitations of our study, language restrictions and methodological shortcomings may affect the generalizability of our findings.

## Introduction

Stroke is one of the leading causes of dysphagia (i.e., unsafe swallowing/swallowing disorders) with incidence rates reaching as high as 80% (Takizawa et al., 2016). This can give rise to a myriad of challenges (e.g., discomfort, frustration, reduced enjoyment of meals, decreased participation in activities of daily living) and health complications (e.g., compromise of nutritional intake, hydration status) including the heightened risk of developing aspiration pneumonia, a condition that can result in fatal outcomes. Considering the significant impact of dysphagia on overall general health and well-being, the need for effective dysphagia treatments is imperative.

Traditional interventions for dysphagia post-stroke include behavioral strategies, such as incorporating compensatory swallowing postures and maneuvers (Logemann, 1991; Langmore and Miller, 1994; Rosenbek, 1995), dietary modifications (e.g., changes in liquid viscosity and, texture and consistency of solid food) (Speyer et al., 2010), sensory stimulation of the oropharynx (Miller, 2008; Martin, 2009; Sdravou et al., 2012), and oral motor stimulation (Kang et al., 2012). In the pursuit of further effective dysphagia treatments, neurostimulation techniques have been extensively explored for post-stroke dysphagia. Two different approaches have been employed so far. One approach concentrates on stimulating the peripheral nervous sensory system through physical stimuli [e.g., tactile-thermal stimulation (Teismann et al., 2009)], chemical stimuli [e.g., capsaicin (Rofes et al., 2013), acid (Logemann et al., 1995)], or electrical stimuli [e.g., transcutaneous neuromuscular or intrapharyngeal electrical stimulation (Jayasekeran et al., 2010)]. The other approach involves the direct stimulation of the cortex, using noninvasive brain stimulation (NIBS) methods such as repetitive transcranial magnetic stimulation (rTMS) and transcranial direct current stimulation (tDCS). The main difference between TMS and tDCS concerns the manner in which they modulate brain activity. TMS applies electromagnetic induction to generate brief, high-intensity magnetic pulses that pass through the skull to induce electrical currents in the brain. These induced currents can either depolarize or hyperpolarize neurons, directly stimulating or inhibiting brain activity in the targeted area. In contrast, tDCS applies a low-intensity, constant direct current to the scalp via electrodes. The current penetrates the skull, modulating the resting membrane potential of neurons. This modulation can influence neuronal excitability thresholds, either increasing or decreasing their likelihood of firing action potentials in response to incoming stimuli. TMS localization depends on coil type and tDCS localization depends on electrode type. For example, a figure-of-eight TMS coil delivers more focused stimulation, directing the majority of the current towards the center of the coil (the “hot spot”). Similarly, high-definition tDCS (HD-tDCS) provides more focused stimulation compared to conventional tDCS setups.

Numerous studies have explored the use of rTMS intervention in post-stroke dysphagia that is either inhibitory (e.g., Kim et al., 2011; Lim et al., 2014) or excitatory (e.g., Park et al., 2013; Momosaki et al., 2014; Lee et al., 2015). However, there is no agreement with regards to the optimal stimulation site (i.e., affected, unaffected or both hemispheres) and stimulation parameters (Jones et al., 2020; Li Y. et al., 2022).

Before conceptualizing new rTMS trials in this field, it is of paramount importance to rigorously assess the available body of literature on the topic. This will enable researchers and clinicians to (i) gain insights into the strengths and limitations of existing intervention strategies and (ii) identify knowledge gaps and areas where further investigation is needed. An attempt was previously made to compile evidence on the effects of rTMS on post-stroke dysphagia (Cheng and Hamdy, 2021) but lacked a systematic approach and an appraisal of previous reviews. Moreover, three commentaries on separate meta-analyses (Li T. et al., 2022; Xie et al., 2022b; Yang and Chang, 2022) have shown that at least three prior systematic reviews suffered from methodological flaws. To comprehensively tackle these limitations, the primary aim of this umbrella review was to appraise the methodological quality of SRs, with and without MAs, that synthesized the findings of randomized controlled trials (RCTs) exploring the effectiveness of rTMS for the management of dysphagia post-stroke. A secondary aim of this study was to evaluate the consistency and reliability of translational implications of rTMS for swallowing recovery after stroke across SRs and MAs.

## Methods

This umbrella review was registered with PROSPERO [ID: CRD42023445474]. The review procedures were established prior to the conduct of this research and the study followed the guidelines outlined in the Cochrane Handbook on Overviews of Reviews (Becker and Oxman, 2008).

We have departed from the pre-registered PROSPERO protocol in terms of the guiding questions for this review, as these questions have been encompassed within the two overarching aims of our study. Also, the original plan was to perform a meta-meta-analysis (MMA) alongside the umbrella review to integrate data from relevant MAs under scrutiny. However, a significant challenge arose during the data extraction process, as the MAs under consideration exhibited substantial overlap of primary studies, raising concerns about potential data duplication and bias. Hence, to avoid erroneous and misleading conclusions, we refrained from performing the MMA.

### Requirements for inclusion

To be considered for inclusion in this study, the SR articles must have been published in English, Greek, French or Italian, as these are the languages spoken by the authors. This umbrella review considered only published SRs using an RCT study design to explore the effectiveness of rTMS for dysphagia rehabilitation post-stroke. Additionally, it encompassed SRs referencing other forms of NIBS methods (e.g., tDCS) using an RCT study design, if they separately explored the specific impact of rTMS on post-stroke dysphagia rehabilitation. Reviews exploring the effects of rTMS on other domains (e.g., language and cognition) were also included if they separately investigated the impact on post-stroke dysphagia rehabilitation. The RCT study design was chosen because it provides a complete picture of the effects of rTMS on post-stroke dysphagia rehabilitation by randomly assigning participants to treatment and control groups, allowing for rigorous comparison. Reviews reporting non-RCTs, case studies, case series or open-label trials, as well as reviews focusing on types of NIBS other than rTMS (e.g., tDCS) were excluded. To qualify for appraisal, the trials reported in the reviews had to adhere to the following requirements:

- be RCTs
- participants of trials were required to have suffered a stroke, regardless of the stage
- the interventions applied in the experimental groups utilized rTMS
- the control groups were mandated to consist of sham/placebo/other dysphagia treatment groups
- the outcome measures included standardized tests of dysphagia assessment (clinical and/or instrumental)

### Search methods, selection of studies and data extraction

The search was conducted on the 14th of May 2023 for all articles published to that date. Five databases were accessed, namely PubMed, Scopus, CINAHL Plus with Full Text, Database of Abstracts of Reviews of Effects (DARE), and Cochrane Database of Systematic Reviews. The search strategies used to access relevant SRs from each database are shown in Supplementary material 1. The references cited in the original entries retrieved were also examined to identify additional potentially relevant studies. Furthermore, as recommended by the AMSTAR 2 (A Measurement Tool to Assess Systematic Reviews) instrument (Shea et al., 2017), the research team made attempts to consult an external content expert.

Using the pre-defined eligibility criteria all identified records were screened, by two independent researchers (AMG & MK), at the title and abstract level, followed by the appraisal of full texts. In the event of conflicts arising during the screening and appraisal processes, the researchers would engage in discussions to reach a final decision; however no conflicts arose in this instance. All procedures were conducted using the Covidence platform, a web-based software specifically designed for efficient collaboration and management (i.e., screening, appraisal, and conflict resolution) of SR processes, leading to a streamlined and organized review process. Authors AMG and PP performed duplicate data extraction and utilized a consensus process for resolving disagreements.

### Assessment of methodological quality

Two authors (AMG & PP) documented the respective rankings of each review independently. Subsequently, these rankings were pooled together and any disparities in opinions throughout the entire procedure were addressed by all three authors until a consensus was ultimately reached for the final ranking of each SR. The methodological conduct of the included studies was evaluated following the AMSTAR 2, an instrument with adequate content validity, inter-rater reliability, and usability (Shea et al., 2017). Shea et al. (2017) have outlined the critical domains, relevant to the conduct of SRs of RCTs, corresponding to specific items (i.e., 2,4,7,9,11,13,15) in the AMSTAR 2 tool. For the purposes of this umbrella review, three additional items (i.e., 1,12,14) were also considered as critical weaknesses.

To investigate the extent of literature overlap among the primary studies included in the SRs, the Graphical Overview of Evidence (GROOVE) (Bracchiglione et al., 2022) was utilized. GROOVE calculates an evidence matrix and a corrected covered area (CCA) and utilizes statistical techniques to gauge overlap, categorizing it as minimal (CCA < 5%), moderate (CCA between 5% and < 10%), substantial (CCA between 10% and < 15%), or extensive (CCA ≥ 15%) (Bracchiglione et al., 2022).

## Results

### Search results

**Figure 1:**
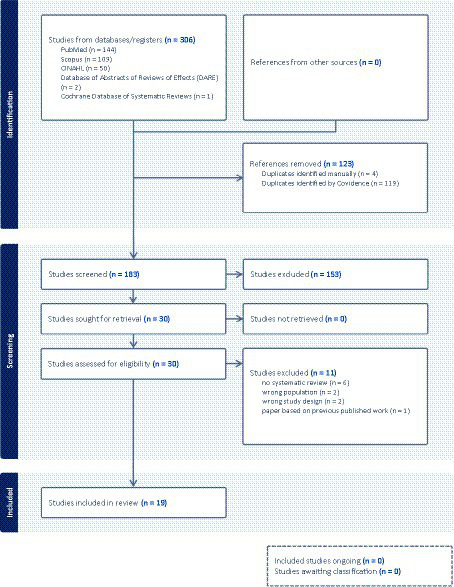
PRISMA screening process flowchart (Page et al., 2021).

Overall 183 studies (after duplicates removal) were identified and screened at the title and abstract level. Thirty were selected for full-text analysis. After applying the eligibility criteria as depicted in Figure 1 of the PRISMA chart, 19 articles were finally included in the review. Studies that were excluded and a justification for their exclusion are reported in Supplementary material 2.

### Characteristics of the systematic reviews

Of the 19 studies included in the analysis, eight SRs investigated the effectiveness of rTMS on post-stroke dysphagia rehabilitation (Liao et al., 2017; Papadopoulou et al., 2018; Yang et al., 2021; Li H. et al., 2022; Qiao et al., 2022; Wen et al., 2022; Xie et al., 2022a; Hsiao et al., 2023) and 11 investigated various therapeutic methods on the topic, including rTMS (Yang et al., 2015; Pisegna et al., 2016; Bath et al., 2018; Chiang et al., 2018; Cheng et al., 2021; Li et al., 2021; Wang et al., 2021; Balcerak et al., 2022; Tan et al., 2022; Zhu and Gu, 2022; Banda et al., 2023). For the specific focus of this umbrella review, from the latter studies, only the information pertaining to the use of rTMS for the treatment of dysphagia post-stroke was analyzed. Two out of the 19 studies performed a SR only (Papadopoulou et al., 2018; Balcerak et al., 2022) and the remaining studies included a MA as well.

The characteristics of the included SRs with/without MAs are reported in Supplementary material 3. Overall, the researchers did not consistently present the characteristics of the included trials, indicating discrepancies in their coverage and analysis. It was not doable to evaluate safety related to rTMS, as report of side effects in many of the included reviews was incomplete or missing.

#### Participants

Analyzing the 19 SRs was challenging in determining the total number of participants for both experimental and control groups. Upon cross-referencing these SRs, it became apparent that the same studies reported participant counts inconsistently across the different reviews. Furthermore, one review (Papadopoulou et al., 2018) lacked any participant related data reporting, and several other reviews either omitted or presented unclear participant data for certain studies. These inconsistencies in participant numbers have made it challenging to determine an accurate cumulative count of participants across the analyzed studies. In response to this challenge, we accessed all primary RCT studies reported in the 19 SRs and extracted their sample sizes. In total, 1,019 participants were included in the primary studies, of which 631 individuals were experimental group participants and 388 individuals were control participants. In addition, certain reviews inadequately reported participants’ demographic characteristics, such as age. The detailed information regarding participant count challenges, inconsistencies, and data reporting issues in the 19 SRs with/without MAs was compiled and is presented in Supplementary materials 4, 5.

#### rTMS protocols in the included studies

The utilization of rTMS varied across studies, with some employing it as a standalone treatment while others combined it with behavioral dysphagia treatment. Within the reviewed trials, a range of rTMS protocols was observed, including frequencies of stimulation spanning 1–10 Hz, varying total session numbers (e.g., 10 sessions vs. 40 sessions), and treatment durations (e.g., 10 min vs. 30 min/day) (see Supplementary material 3). After excluding SRs without data on TMS localization, we identified a consistent focus on the pharyngeal, mylohyoid, tongue, and esophageal motor cortex, targeted contralaterally, ipsilaterally, or bilaterally. The emphasis on these four brain areas underscores their fundamental involvement in regulating swallowing function. Importantly, there was also inconsistency across the studies in the reporting of critical rTMS parameters, such as localization methods and threshold estimations. This lack of uniformity was further amplified by certain reviews that failed to include these essential details.

#### Outcome measures

In the majority of trials, dysphagia was evaluated using a combination of tools, including bedside clinical evaluation of swallowing and/or instrumental evaluation methods such as videofluoroscopy (VFS). The information on outcome measures used is reported in Supplementary material 3.

#### Collective effects of rTMS on post-stroke dysphagia

Two out of the 19 studies were SRs without MAs as follows: Balcerak et al. (2022) reported at least some therapeutic benefits of rTMS on post-stroke dysphagia, suggesting that rTMS may be a promising therapeutic option for dysphagia post-stroke. Papadopoulou et al. (2018) did not provide sufficient details regarding the study design, total number of participants, chronicity, type of stroke, and outcome measures used in the included studies. Furthermore, not all reported information reported was relevant to dysphagia (e.g., including language data), which may have been unintentional, but complicates the analysis of the research findings.

The results of each of the 17 MAs can be found in Supplementary material 3. All MAs provided at least some significant evidence favouring the effectiveness of rTMS for improving swallowing outcomes in post-stroke dysphagia. Notably, most studies provided evidence of heterogeneity of the included studies, as well as possible publication bias. The collective findings from the MAs regarding the effects of rTMS on post-stroke dysphagia have been classified into five distinct categories based on specific features as reported below:

##### The impact on swallowing ability

Regarding the impact on swallowing ability, 12 studies reported large effects of rTMS compared to control conditions (Yang et al., 2015; Liao et al., 2017; Chiang et al., 2018; Li et al., 2021; Wang et al., 2021; Li H. et al., 2022; Qiao et al., 2022; Tan et al., 2022; Wen et al., 2022; Xie et al., 2022a; Zhu and Gu, 2022; Banda et al., 2023), two studies found moderate effects (Pisegna et al., 2016; Cheng et al., 2021), one study found marginal effects (Yang et al., 2021) and one study reported that rTMS improved swallowing ability -however, the finding could be due to chance, considering no subgroup differences and heterogeneity (Bath et al., 2018).

##### The stimulation site

With respect to the site of stimulation (contralateral, ipsilateral, or bilateral), one study reported that stimulation of both hemispheres may lead to better overall swallowing gains in comparison to stimulation of the ipsilesional/contralesional hemisphere (Xie et al., 2022a), one study reported better swallowing outcomes when the contralateral or bilateral hemispheres were stimulated (Liao et al., 2017), one study found that HF ipsilesional rTMS initially showed the strongest positive swallowing effect (Cheng et al., 2021) but this effect disappeared when a high-risk of bias (RoB) study was excluded. Interestingly, three studies (Li H. et al., 2022; Qiao et al., 2022; Wen et al., 2022) did not find significant differences between subgroups according to stimulation site, which included the affected hemisphere, unaffected hemisphere, or both hemispheres.

##### The stimulation frequency

In the context of examining stimulation frequency, three studies reported no difference between low frequency (LF) and high frequency (HF) (Yang et al., 2021; Qiao et al., 2022; Zhu and Gu, 2022). However, a significant revelation came from sensitivity analyses within the Qiao et al. (2022) study, where a considerably higher effect size was found within the LF group. Conversely, one study (Cheng et al., 2021) found that HF ipsilesional rTMS initially showed the strongest positive effect but this effect disappeared when a high-RoB study was excluded, two studies reported that HF effects were significantly greater than LF effects (Liao et al., 2017; Wen et al., 2022), and one study reported that LF-rTMS treatment produced better effects on overall swallowing function than HF-rTMS treatment (Xie et al., 2022a).

##### Long-lasting treatment effects

One study reported improvements at one- and two-months post-treatment (Li H. et al., 2022), one study did not find significant effects of treatment during the follow-up period (Tan et al., 2022), and one study reported moderate effects for early and intermediate follow-up, but no significant effects for late follow-up (Cheng et al., 2021).

##### Treatment effects on specific outcome measures

Regarding pharyngeal transit time (PPT) and oral transit time (OTT), one study did not find improvements (Banda et al., 2023). With regards to penetration/aspiration, two studies found large effects of rTMS (Li et al., 2021; Xie et al., 2022a), two studies did not report any benefits (Bath et al., 2018; Banda et al., 2023), and one study reported no effects on HF-rTMS applied on the ipsilesional cortex and a medium effect of LF rTMS applied on the contralesional cortex immediately after intervention but not at 4-week follow-up (Hsiao et al., 2023). Importantly, the study of Xie et al. (2022a), after performing a subgroup analysis of penetration/aspiration, found that bilateral stimulation may produce better therapeutic effects on overall swallowing function in comparison to ipsilesional/contralesional stimulation and that LF-rTMS treatment produced better effects on overall swallowing function than HF-rTMS treatment.

With reference to the standardized swallowing assessment (SSA), one study reported large effects of rTMS (Li et al., 2021), and one study reported medium effects by HF rTMS applied on the ipsilesional cortex immediately after intervention and at 4-week follow-up and medium effects by LF rTMS applied on the contralesional cortex immediately after intervention and a large effect at 4-week follow-up (Hsiao et al., 2023).

In one study (Li et al., 2021), all individual outcome-focused meta-analyses revealed an advantage of rTMS therapy over control interventions for dysphagia based on the dysphagia outcome severity scale (DOSS), the functional dysphagia scale (FDS) and the water swallow test (WST).

One study found no effects of rTMS on participant fatality (Bath et al., 2018), and one study found that subgroup analyses for race (Asians/Caucasians) indicated significant benefits of rTMS on swallowing for Asians (insignificant for Caucasians but with k = 3 and high error) (Zhu and Gu, 2022). Notably, in the study of Xie et al. (2022a), a subgroup analysis of the Barthel Index (BI) scale, showed significant improvements in activities of daily living for the rTMS group compared to the control group. There were no differences in dropout rates between the rTMS group and the control group, and no differences in adverse effects were observed between those two groups. In particular, the study by Qiao et al. (2022) that examined rTMS response concerning treatment duration (number of sessions and the amount of stimulation per session), stroke chronicity, and age, proposed that treatment durations exceeding 5 days yielded a higher effect size. Regarding stimulation duration, both ≤10 min and > 10 min showed a higher effect than the control conditions. Concerning stroke chronicity, the introduction of rTMS in the subacute phase (<60 days post-stroke), resulted in significantly greater benefits compared to the control conditions, whereas rTMS applied during the recovery phase (>60 days post-stroke) did not surpass the control. In the age subgroup analysis, significant positive effects were found for all age groups compared to control conditions.

### Evaluation of the quality of conduct of the systematic reviews

Table 1 reports on the assessment of methodological quality for the 19 studies conducted using the AMSTAR 2 tool (Shea et al., 2017). Two studies (Tan et al., 2022; Banda et al., 2023), received low quality ratings, while the remaining (17), were rated with critically low quality. Furthermore, nine SRs did not incorporate mean scores and standard deviations for both the rTMS and control groups in their MAs (see Supplementary material 4). Notably, different means, standard deviations, and sample sizes, were reported across the various MAs for the same primary study, in many cases (see Supplementary materials 4, 5).

**Table 1:** Methodological quality and overall confidence ratings of the 19 systematic reviews (with/without meta-analyses) using the AMSTAR 2 checklist.

Study	AMSTAR 2 checklist items (Shea et al., 2017)																Overall quality
	1	2	3	4	5	6	7	8	9	10	11	12	13	14	15	16	
Balcerak et al. (2022)	Y	N	N	N	Y	Y	N	N	Y	N	-	-	Y	N	-	Y	Critically low
Papadopoulou et al. (2018)	N	N	N	N	N	N	N	N	N	N	-	-	N	N	-	Y	Critically low
Banda et al. (2023)	Y	Y	N	P	N	Y	N	Y	Y	N	Y	Y	Y	Y	Y	Y	Low
Hsiao et al. (2023)	Y	N	N	P	Y	Y	N	P	Y	N	Y	N	N	Y	N	Y	Critically low
Li H. et al. (2022)	Y	N	N	P	N	Y	N	P	Y	N	N	N	N	Y	N	Y	Critically low
Qiao et al. (2022)	Y	P	N	P	N	Y	N	Y	Y	N	Y	N	Y	Y	Y	Y	Critically low
Tan et al. (2022)	Y	Y	N	Y	Y	Y	Y	Y	Y	N	Y	N	Y	Y	Y	Y	Low
Wen et al. (2022)	Y	P	N	P	Y	Y	N	P	Y	N	N	N	N	Y	N	Y	Critically low
Xie et al. (2022a)	Y	N	N	N	Y	Y	N	P	Y	N	Y	N	N	Y	Y	Y	Critically low
Zhu and Gu (2022)	N	N	N	P	Y	N	N	N	N	N	N	N	N	N	Y	Y	Critically low
Cheng et al. (2021)	Y	N	N	P	Y	Y	N	Y	Y	N	Y	Y	N	Y	N	Y	Critically low
Li et al. (2021)	Y	N	N	P	Y	Y	N	N	Y	N	Y	N	N	N	Y	Y	Critically low
Wang et al. (2021)	Y	N	N	P	N	Y	N	P	Y	N	Y	N	N	N	N	Y	Critically low
Yang et al. (2021)	Y	N	N	N	Y	Y	N	N	Y	N	Y	N	Y	Y	N	Y	Critically low
Bath et al. (2018)	Y	N	Y	Y	Y	Y	Y	P	Y	N	Y	N	Y	N	N	Y	Critically low
Chiang et al. (2018)	Y	N	N	N	N	Y	Y	Y	Y	N	N	N	N	N	Y	N	Critically low
Liao et al. (2017)	N	N	N	N	Y	Y	N	P	Y	N	Y	N	N	Y	N	Y	Critically low
Pisegna et al. (2016)	N	N	N	Y	Y	N	N	Y	Y	N	Y	Y	Y	Y	Y	Y	Critically low
Yang et al. (2015)	Y	N	N	P	Y	N	N	Y	Y	N	Y	N	N	N	N	Y	Critically low

Key: Yes (Y), all information was provided; No (N), no information is provided; Partial Yes (P), partial information was provided.

**Figure 2:**
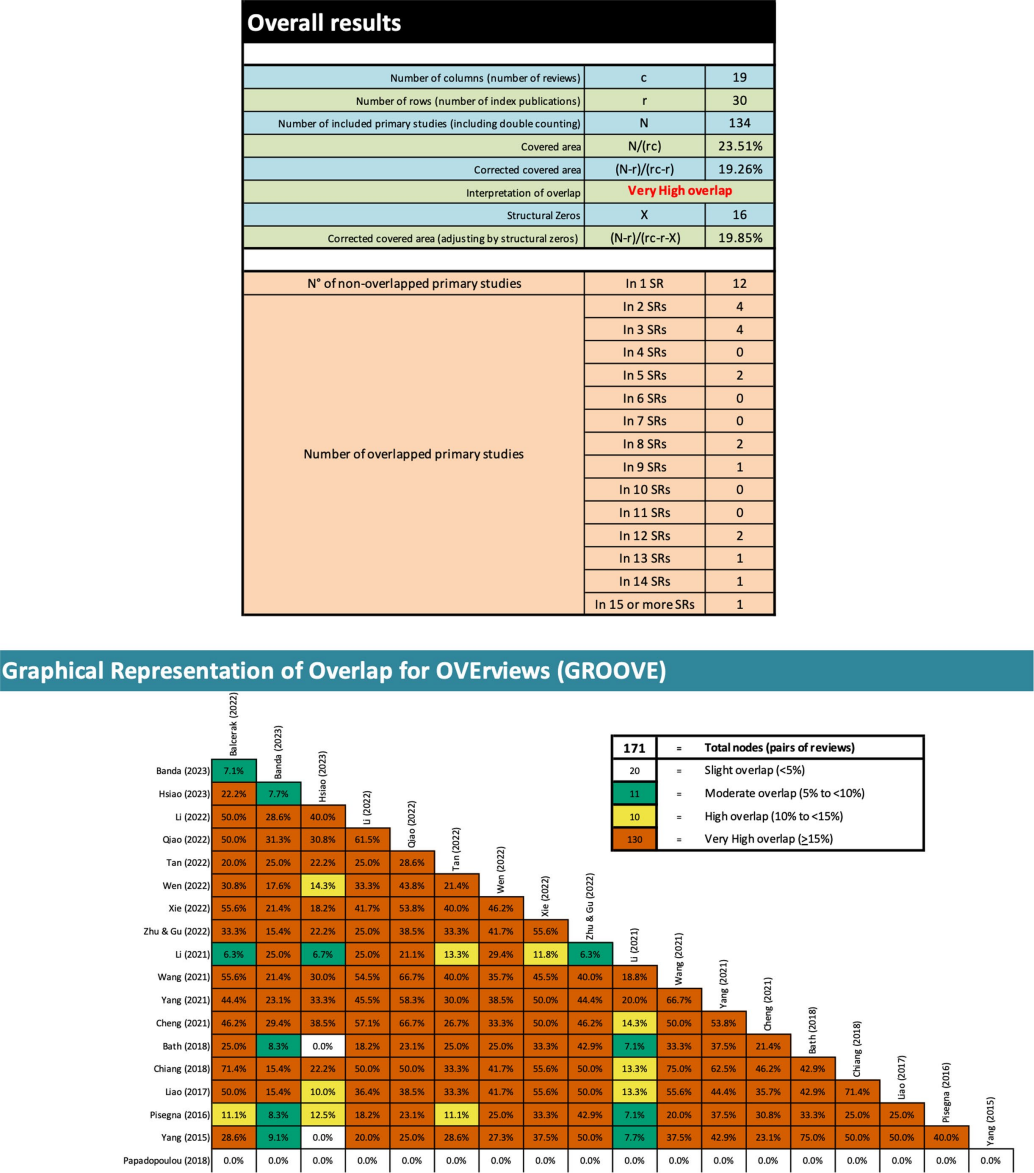
Overlapping of the included reviews.

Through GROOVE, 171 nodes were found, indicating very high overlap across the reviews (Figure 2). Twelve primary studies were not overlapping (i.e., appeared only in a single review), five appeared in at least 12 of the reviews, while the remaining 13 appeared in at least two reviews, but in no more than nine reviews. Structural missingness was used for the study by Papadopoulou et al. (2018) because it was unclear which primary studies were included in the review (for all primary studies published until 2018).

## Discussion

The aim of this umbrella review was twofold. First, to rigorously assess the quality of evidence regarding the utilization of rTMS for the treatment of post-stroke dysphagia, as documented in prior SRs and MAs. Second, to evaluate the consistency and reliability of translational implications of rTMS for swallowing recovery after stroke across these SRs and MAs. This comprehensive approach was undertaken to provide a reliable assessment of rTMS’s potential efficacy in treating post-stroke dysphagia.

Among the 19 included studies, two studies (Tan et al., 2022; Banda et al., 2023) received low quality ratings, while the rest (17) were rated with critically low quality. In many studies we identified additional methodological flaws, which raise questions about the reliability of the results. For example, nine studies (Liao et al., 2017; Bath et al., 2018; Chiang et al., 2018; Cheng et al., 2021; Wang et al., 2021; Yang et al., 2021; Balcerak et al., 2022; Zhu and Gu, 2022; Banda et al., 2023) did not incorporate mean scores and standard deviations for both the rTMS and control groups in their MAs. Also, different means, standard deviations, and sample sizes were reported across the various MAs for the same primary study, in many cases. Moreover, in numerous cases, multiple effect sizes were included from the same primary studies, raising the issue of violating the independence of the effect sizes. Some of the studies exhibited poor descriptions of participant demographics and/or rTMS parameters (Bath et al., 2018; Papadopoulou et al., 2018; Yang et al., 2021; Balcerak et al., 2022; Banda et al., 2023). A significant methodological issue that deserves attention is the inclusion of both stroke and traumatic brain injury (TBI) participants in several SRs (Pisegna et al., 2016; Liao et al., 2017; Bath et al., 2018; Cheng et al., 2021; Yang et al., 2021; Qiao et al., 2022; Wen et al., 2022; Xie et al., 2022a; Zhu and Gu, 2022; Hsiao et al., 2023). Notably, this issue arises from the fact that one primary study (Kim et al., 2011), which included both stroke and TBI patients, was incorporated in many of these SRs. This drawback was particularly noteworthy because the focus of the SRs and their respective MAs was on assessing the effects of rTMS specifically in the context of post-stroke dysphagia. Even though the number of TBI participants may have been small (*n* = 2), their presence in the analyses raises concerns about the validity of the results. Another significant flaw was observed in certain SRs where a study combined rTMS with Neuromuscular Electrical Stimulation (NMES), and no standalone rTMS group was included as an experimental group (Zhang et al., 2019). Since NMES is a recognized treatment in its own right, this combination may have introduced confounding variables that could have potentially distorted the results of the SRs (Cheng et al., 2021; Li H. et al., 2022; Hsiao et al., 2023).

With regards to our second aim, which involved evaluating the consistency and reliability of translational implications of rTMS for swallowing recovery after stroke, we discovered a consistent presence of at least some significant evidence supporting the effectiveness of rTMS in enhancing swallowing outcomes for individuals with dysphagia post-stroke in all SRs and MAs. Yet, it is important to recognize that most of these studies also identified heterogeneity among their included studies and potential publication bias, indicating the need for cautious interpretation of the findings. The collective findings from the MAs regarding the effects of rTMS on post-stroke dysphagia have been classified into distinct groups based on the information reported as follows: (i) on the impact on swallowing ability, (ii) on stimulation site, (iii) on stimulation frequency, (iv) on the long-lasting effects of treatment and (v) on the treatment effects as measured by specific outcome measures. With regards to the impact of rTMS on swallowing ability, all studies but one (Bath et al., 2018) indicate that it is an effective treatment option. The research findings regarding the impact of stimulation site on swallowing improvement revealed a notable level of inconsistency that underscores the complexity of this aspect of rTMS research in, at least, the context of post-stroke dysphagia. Studies exploring the impact of stimulation frequency on swallowing function revealed inconsistent results as well. This contradiction highlights the lack of consensus on the optimal stimulation frequency for improving post-stroke dysphagia. Studies investigating the long-term effects of treatment yielded conflicting findings. Some reported improvements at various follow-up intervals, while others found no significant effects during specific periods. The obvious lack of consistency in research methods on assessing the long-term effects of rTMS effects on post-stroke dysphagia, is compounded by the absence of follow-up data. The assessment of rTMS effects on specific outcome measures, as scrutinized by various SRs, exposed significant inconsistencies. These SRs differed in the outcome measures they prioritized and analyzed, contributing to a lack of uniformity in their research focus and findings and making direct comparisons between studies challenging. This variability underscores the importance of establishing a standardized set of outcome measures within the field of rTMS research for post-stroke dysphagia. Such standardization would facilitate more comprehensive and meaningful assessments of treatment effectiveness, providing valuable insights for both research and clinical practice.

According to the principles of evidence-based medicine, SRs are typically regarded as having the highest level of credibility. However, in the context of rTMS for post-stroke dysphagia rehabilitation, the number of SRs significantly outweighs the number of primary RCTs. This has two critical implications. Firstly, this wealth of SRs leads to a considerable overlap evident from our GROOVE analysis (Figure 2). The considerable overlap in the case of a meta-MA may lead to an overly precise but inaccurate estimate of the effectiveness of the intervention under investigation, due to redundant findings biasing the estimate towards an overrepresented effect size (Ioannidis, 2016; Bracchiglione et al., 2022). While the practice of repeatedly including the same studies in MAs serves the purpose of consolidating evidence and increasing the statistical strength of the findings, it also carries the risk of reinforcing specific patterns of findings within the results and placing undue emphasis on outcomes, that may be misleading. Secondly, this imbalance in the number of SRs versus primary research can lead to a potential emphasis on synthesized evidence at the expense of primary studies. This is particularly concerning given the low methodological quality of many of these SRs, as rated by AMSTAR 2 in this study. This finding raises concerns about the reliability of conclusions drawn from the MAs within these SRs. Overall, the current state of evidence suggests that the efficacy of rTMS on treating post-stroke dysphagia is not reported in a consistent and/or reliable manner to inform clinical practice. It appears, despite the large number of individual studies on the topic, that the ‘gold-standard’ remains elusive to researchers hindering rTMS applications in real world settings to treat post-stroke dysphagia.

Based on the findings, we propose several recommendations. First, caution is warranted when interpreting the results of current published SRs on the effectiveness of TMS for post-stroke dysphagia management, given the current state of evidence. Second, there is an urgent need for further large-scale RCTs to establish the evidence on the effectiveness of rTMS as a post-stroke dysphagia treatment method. In light of the inconsistencies observed in previous research regarding the long-term effects of rTMS on post-stroke dysphagia, it becomes evident that future studies should prioritize the robust collection of extended follow-up data with meaningful endpoints (i.e., long-term disability, pneumonia, and mortality). Third, the development of high-quality SRs that adhere to AMSTAR 2 guidelines is crucial. Regarding the methodology of future MAs, if significant heterogeneity exists, we recommend conducting moderator and/or exploratory analyses to investigate the possible causes, and thus enhance the quality of evidence. It is proposed that future MAs report or provide the extracted data as well as the methodological details of standardizing and pooling the effect sizes. Further, MAs need to address the possible violation of effect size independence, for example, by adding an additional level to the MA model, so that between-study variance is accounted for. Moreover, during the research planning phase, it is advisable to register or publish research plans in platforms like PROSPERO. Future research efforts should also prioritize standardizing TMS procedures, to include localization, intensity, operation frequency, and timing to enhance the reproducibility and comparability of findings across studies.

Concerning the limitations of our study, language restrictions and methodological limitations may affect the generalizability of our findings. In particular, language restrictions may have resulted in the exclusion of relevant studies or introduced a language bias in our review, potentially limiting the representativeness of our findings. Furthermore, the subjective nature of the AMSTAR 2 can introduce a potential source of bias since different reviewers may interpret and apply its criteria differently, which may affect the overall assessment of the studies. To mitigate the subjectivity inherent in the AMSTAR 2 assessment tool, we employed a dual-assessor approach, to enhance the objectivity and reliability of our quality assessments. Also, it is important to acknowledge that the definition of critical items in the AMSTAR 2 can be somewhat arbitrary. However, this particular concern may not significantly affect the study findings since the majority of studies evaluated received low ratings across most of the AMSTAR 2 criteria. Finally, the omission of non-RCTs, case studies, case series, or open-label trials in our study could narrow the scope of evidence examined. Nevertheless, it is common for SRs to consider mainly RCTs due to their rigorous methodology. Therefore, while this exclusion may be seen as a limitation, it follows the standard practice of emphasizing RCTs to ensure the reliability of the synthesized evidence.

Recognizing the existing knowledge within the stroke medicine community with regards to the non-standard application of rTMS for dysphagia rehabilitation post-stroke, our umbrella review highlights the critical role of continued investigation into rTMS for dysphagia post-stroke to elucidate possible therapeutic benefits and address current limitations in the evidence base. Future research should prioritize large-scale, high-quality primary rTMS intervention studies for post-stroke dysphagia management. Future SRs need to address the inconsistencies and methodological limitations within the existing body of research. This will be invaluable for the development of a reporting guideline in the field, that will inform clinical decision-making and advance patient care in this critical area of stroke rehabilitation.

## Author contributions

AG: Writing – review & editing, Writing – original draft, Visualization, Methodology, Investigation, Formal analysis, Data curation, Conceptualization. PP: Writing – review & editing, Visualization, Methodology, Formal analysis, Data curation. MK: Writing – review & editing.
