# Supplementary material for: The effectiveness of transcranial magnetic stimulation for dysphagia in stroke patients: an umbrella review of systematic reviews and meta-analyses
Source: Front Hum Neurosci. 2024 Mar 14;18:1355407. doi: 10.3389/fnhum.2024.1355407 (PMC10972992; doi:10.3389/fnhum.2024.1355407)
Supplement: Supplementary file 1 [file Data_Sheet_1.docx]

**Supplementary Material 1**

Search strategies used to access relevant systematic reviews from each database on the application of transcranial magnetic stimulation (TMS) for post-stroke dysphagia rehabilitation.

**PubMed**

("deglutition disorders"[MeSH Terms] OR ("deglutition"[All Fields] AND "disorders"[All Fields]) OR "deglutition disorders"[All Fields] OR "dysphagia"[All Fields] OR "dysphagias"[All Fields] OR ("deglutition disorders"[MeSH Terms] OR ("deglutition"[All Fields] AND "disorders"[All Fields]) OR "deglutition disorders"[All Fields] OR ("swallowing"[All Fields] AND "disorders"[All Fields]) OR "swallowing disorders"[All Fields]) OR ("deglutition disorders"[MeSH Terms] OR ("deglutition"[All Fields] AND "disorders"[All Fields]) OR "deglutition disorders"[All Fields])) AND ("stroke"[MeSH Terms] OR "stroke"[All Fields] OR "strokes"[All Fields] OR "stroke s"[All Fields] OR ("stroke"[MeSH Terms] OR "stroke"[All Fields] OR ("cerebrovascular"[All Fields] AND "accident"[All Fields]) OR "cerebrovascular accident"[All Fields]) OR ("stroke"[MeSH Terms] OR "stroke"[All Fields] OR "cva"[All Fields])) AND ((("neuromodulate"[All Fields] OR "neuromodulating"[All Fields] OR "neuromodulation"[All Fields] OR "neuromodulations"[All Fields] OR "neuromodulative"[All Fields] OR "neurotransmitter agents"[Pharmacological Action] OR "neurotransmitter agents"[MeSH Terms] OR ("neurotransmitter"[All Fields] AND "agents"[All Fields]) OR "neurotransmitter agents"[All Fields] OR "neuromodulator"[All Fields] OR "neuromodulators"[All Fields]) AND "non"[All Fields] AND ("invasibility"[All Fields] OR "invasible"[All Fields] OR "invasion"[All Fields] OR "invasions"[All Fields] OR "invasive"[All Fields] OR "invasively"[All Fields] OR "invasiveness"[All Fields] OR "invasives"[All Fields] OR "invasivity"[All Fields])) OR ("symp theory model simul"[Journal] OR "tms"[All Fields] OR ("transcranial magnetic stimulation"[MeSH Terms] OR ("transcranial"[All Fields] AND "magnetic"[All Fields] AND "stimulation"[All Fields]) OR "transcranial magnetic stimulation"[All Fields]) OR ("transcranial magnetic stimulation"[MeSH Terms] OR ("transcranial"[All Fields] AND "magnetic"[All Fields] AND "stimulation"[All Fields]) OR "transcranial magnetic stimulation"[All Fields] OR "rtms"[All Fields])) OR ("transcranial direct current stimulation"[MeSH Terms] OR ("transcranial"[All Fields] AND "direct"[All Fields] AND "current"[All Fields] AND "stimulation"[All Fields]) OR "transcranial direct current stimulation"[All Fields] OR "tdcs"[All Fields] OR ("transcranial direct current stimulation"[MeSH Terms] OR ("transcranial"[All Fields] AND "direct"[All Fields] AND "current"[All Fields] AND "stimulation"[All Fields]) OR "transcranial direct current stimulation"[All Fields])))

**SCOPUS**

TITLE-ABS-KEY

( ( ( dysphagia  OR  swallowing  AND disorders  OR  deglutition  AND disorders )

AND  ( stroke  OR  cerebrovascular  AND accident  OR  cva ) )  AND  ( ( neuromodulation  AND non  AND invasive )  OR  ( tms  OR  transcranial  AND magnetic  AND stimulation

OR  rtms )  OR  ( tdcs  OR  transcranial  AND direct  AND current  AND stimulation ) ) )

**CINAHL Plus with Full Text**

| **#** | **Query** | **Limiters/Expanders** | **Last Run Via** |
| --- | --- | --- | --- |
| **S1** | (( dysphagia or swallowing disorders or deglutition disorders ) AND ( stroke or cerebrovascular accident or cva )) AND (( neuromodulation non invasive ) OR ( tms or transcranial magnetic stimulation or rtms ) OR ( tdcs or transcranial direct current stimulation )) | Expanders - Apply equivalent subjects Search modes - Boolean/Phrase | Interface - EBSCOhost Research Databases Search Screen - Advanced Search  Database - CINAHL Plus with Full Text |

**Database of Abstracts of Reviews of Effects (DARE)**

(( dysphagia or swallowing disorders or deglutition disorders ) AND ( stroke or cerebrovascular accident or cva )) AND (( neuromodulation non invasive ) OR ( tms or transcranial magnetic stimulation or rtms ) OR ( tdcs or transcranial direct current stimulation ))

**Cochrane Database of Systematic Reviews**

(( dysphagia or swallowing disorders or deglutition disorders ) AND ( stroke or cerebrovascular accident or cva )) AND (( neuromodulation non invasive ) OR ( tms or transcranial magnetic stimulation or rtms ) OR ( tdcs or transcranial direct current stimulation ))
