# Supplementary material for: The effectiveness of transcranial magnetic stimulation for dysphagia in stroke patients: an umbrella review of systematic reviews and meta-analyses
Source: Front Hum Neurosci. 2024 Mar 14;18:1355407. doi: 10.3389/fnhum.2024.1355407 (PMC10972992; doi:10.3389/fnhum.2024.1355407)
Supplement: Supplementary file 2 [file Data_Sheet_2.docx]

**Supplementary Material 2**

Research articles not included in the analysis, along with the reasoning behind their exclusion.

| **References (in chronological order)** | **Reasons for exclusion** |
| --- | --- |
| Li, Y., Chen, K., Wang, J., Lu, H., Li, X., Yang, L., Zhang, W., Ning, S., Wang, J., Sun, Y., Song, Y., Zhang, M., Hou, J., & Shi, H. (2022). Research progress on transcranial magnetic stimulation for post-stroke dysphagia. *Frontiers in behavioral neuroscience*, *16*, 995614.  https://doi.org/10.3389/fnbeh.2022.995614 | Not a systematic review |
| Liu, J., Zhuo, H., & Sun, M. (2022). Rehabilitation of Post-Stroke Swallowing Dysfunction with Repeated Transcranial Magnetic Stimulation RTMS Based on Tomographic Images. *Contrast media & molecular imaging*, *2022*, 1118745.  https://doi.org/10.1155/2022/1118745 | Wrong study design |
| Cheng, I., & Hamdy, S. (2021). Current perspectives on the benefits, risks, and limitations of noninvasive brain stimulation (NIBS) for post-stroke dysphagia. *Expert review of neurotherapeutics*, *21*(10), 1135–1146.  https://doi.org/10.1080/14737175.2021.1974841 | Not a systematic review |
| Eskildsen, S. J., Poulsen, I., Jakobsen, D., Riberholt, C. G., & Curtis, D. J. (2021). Scoping review to identify and map non-pharmacological, non-surgical treatments for dysphagia following moderate-to-severe acquired brain injury. *BMJ open*, *11*(12), e053244.  https://doi.org/10.1136/bmjopen-2021-053244 | Wrong population |
| Kim, W. J., Rosselin, C., Amatya, B., Hafezi, P., & Khan, F. (2020). Repetitive transcranial magnetic stimulation for management of post-stroke impairments: An overview of systematic reviews. *Journal of rehabilitation medicine*, *52*(2), jrm00015. https://doi.org/10.2340/16501977-2637 | Not a systematic review |
| Bath, P. M., Lee, H. S., & Everton, L. F. (2019). Swallowing Therapy for Dysphagia in Acute and Subacute Stroke. *Stroke, 50,* e46–e47.  https://doi.org/10.1161/STROKEAHA.118.024299 | Paper based on previous published work (doi: 10.1002/14651858.CD000323) |
| Dionísio, A., Duarte, I. C., Patrício, M., & Castelo-Branco, M. (2018). Transcranial Magnetic Stimulation as an Intervention Tool to Recover from Language, Swallowing and Attentional Deficits after Stroke: A Systematic Review. *Cerebrovascular diseases (Basel, Switzerland)*, *46*(3-4), 178–185.  https://doi.org/10.1159/000494213 | Wrong study design |
| Simons, A., & Hamdy, S. (2017). The Use of Brain Stimulation in Dysphagia Management. *Dysphagia*, *32*(2), 209–215.  https://doi.org/10.1007/s00455-017-9789-z | Not a systematic review |
| Wang, Z., Song, W. Q., & Wang, L. (2017). Application of noninvasive brain stimulation for post-stroke dysphagia rehabilitation. *The Kaohsiung journal of medical sciences*, *33*(2), 55–61.  https://doi.org/10.1016/j.kjms.2016.11.007 | Not a systematic review |
| Doeltgen, S. H., Bradnam, L. V., Young, J. A., & Fong, E. (2015). Transcranial non-invasive brain stimulation in swallowing rehabilitation following stroke--a review of the literature. *Physiology & behavior*, *143*, 1–9.  https://doi.org/10.1016/j.physbeh.2015.02.025 | Wrong population |
| Hamdy, S. (2010). Role of Neurostimulation and neuroplasticity in the rehabilitation of dysphagia after stroke. SIG 13 Perspectives on Swallowing and Swallowing Disorders. *Dysphagia,* 19, 3–9.  https://doi.org/10.1044/sasd19.1.3 | Not a systematic review |
