# Supplementary material for: The effectiveness of transcranial magnetic stimulation for dysphagia in stroke patients: an umbrella review of systematic reviews and meta-analyses
Source: Front Hum Neurosci. 2024 Mar 14;18:1355407. doi: 10.3389/fnhum.2024.1355407 (PMC10972992; doi:10.3389/fnhum.2024.1355407)
Supplement: Supplementary file 3 [file Data_Sheet_3.docx]

| **Systematic Reviews with/without  meta-analyses (references)** | **Total number of studies & participants** | **Chronicity & type  of stroke** | **Interventions** | **Outcome  measures** | **Follow-up** | **Main findings** | **Meta-analysis results (SMD [95% CI])** | **Sources  of funding** |
| --- | --- | --- | --- | --- | --- | --- | --- | --- |
| 1. Balcerak et al. (2022) without meta-analysis | – 6 studies with  170 participants in total | – Chronicity of stroke not clear: maybe within 3 months after ischemic stroke – Ischemic stroke | – Similar concept of applying rTMS over the affected motor cortex across all studies – Different types  of rTMS treatment between studies (e.g., 1-10 Hz, rTMS alone vs rTMS with standard therapy) | – Variation  across studies – All studies except two used clinical dysphagia assessment only – Only two studies used both clinical and instrumental (VFS) dysphagia assessment | – Not reported | – All of the 6 included trials reported at least some  therapeutic effects of rTMS on post-stroke dysphagia | – N/A | – Internal resources of the Department of Neurology  and Stroke Center, Cantonal Hospital St. Gallen, St. Gallen, Switzerland |
| 2. Papadopoulou et al. (2018) without  meta-analysis | – 26 studies;  the total number of participants is not explicitly mentioned | – The review does  not specify the chronicity and types of stroke in the included trials | – The authors provide incomplete and inconsistent reporting of intervention protocols across the reported trials | – According to the authors:  "Only studies in which a valid tool (namely, video fluoroscopy swallowing study) was used for the assessment of dysphagia were included in the review." (p.392) | – Not reported | – The authors report that "Overall, the results warrant rTMS as an instrumental, noninvasive method for the rehabilitation of dysphagic patients, and further studies in the future may solidify its usefulness as well as its long‐term and far‐reaching effects." (p.395) | – N/A | – No financial support |
| 3. Banda et al. (2023) with meta-analysis | – 9 studies with 255 participants in total | – Acute, sub-acute & crhonic  – Ischemic & hemorrhagic stroke | – TMS applied as a standalone vs an adjuvant therapy – The number of sessions ranged from 10 to 40  – The duration of sessions ranged from 10 to 30 min/day – The site of stimulation was not reported | – Primary (i.e., swallowing function) and secondary outcome measures (PTT, risk of penetration/aspiration, OTT)  – Variation  across studies (i.e., clinical dysphagia assessment only vs clinical and instrumental evaluation vs instrumental evaluation only)  – Variation in tools | – Limited, up to 2 months | – rTMS with TDT and rTMS alone were effective therapies for better swallowing function – rTMS with TDT and rTMS alone revealed no difference in reducing PTT, OTT or aspiration/penetration | Swallowing function:  – rTMS+TDT: 3.32 [1.18, 5.47] – rTMS: 0.86 [−0.71, 2.42]   Reduction of PTT:  – rTMS+TDT: −0.44 [−0.67, −0.21]  – rTMS: −0.22, [−0.59, 0.16]   Reduction of OTT:  – rTMS: −0.51 [−0.93, −0.08]   Reduction of aspiration / penetration: – rTMS+TDT: −2.65 [−7.68, 2.38]  – rTMS: −0.79 [−2.67, 1.08] | – No financial support |
| 4. Hsiao et al. (2023) with meta-analysis | – 6 studies with 158 participants in total | – No details on stroke chronicity  – Ischemic & hemorrhagic stroke | – The duration of treatment ranged from 5 to 10 days – The number of pulses ranged from 900 to 1200 ranged from 5 to 10 days – The stimulation frequency varied between low and high (i.e., 1 vs ≥3 Hz) – The site of stimulation was either the ipsilesional or contralesional mylohyoid cortex, except one (i.e., contralesional pharyngeal cortex) – The RMT ranged from 80-110% | – All studies used both clinical and instumrental evaluation of swallowing but the tools varied  across studies | – Limited, up to 3 months | PAS scores: – No effect by HF rMTS applied on the ipsilesional cortex  – Medium effect by LF rTMS applied on the contralesional cortex immediately after intervention but not at 4-week follow-up  SSA scores: – Medium effect by HF rTMS applied on the ipsilesional cortex immediately after intervention and at 4-week follow-up – Medium effect by LF rTMS applied on the contralesional cortex immediately after intervention and high effect at 4-week follow-up | PAS scores: – HF rTMS ipsilesional, immediate effects: 0.25 [-0.36, 0.86] – HF rTMS ipsilesional, 3-week follow-up: 0.37 [-0.47, 1.22] – LF rTMS contralesional, immediate effects: -0.60 [-1.19, -0.01] – LF rTMS contralesional, 4-week follow-up: -0.19 [-0.71, 0.33]  SSA scores: – HF rTMS ipsilesional, immediate effects: -0.61 [-1.14, -0.08] – HF rTMS ipsilesional, 4-week follow-up: -0.74 [-1.27, -0.21] – LF rTMS contralesional, immediate effects: -0.59 [-1.12, -0.06] – LF rTMS contralesional, 4-week follow-up: -0.92 [-1.48, -0.37] | – National Research Foundation  of Korea grand funded by the Korean government  (grant no.  NRF-2019M3E5D1A02068106) |
| 5. Li et al. (2022) with meta-analysis | – 9 studies with 393 participants in total | – Stroke chronicity: < 6 months post-stroke  – Unilateral ischemic / hemorrhagic stroke | – The duration of treatment was 5 or 10 days – In 4 studies the duration of each session was reported (i.e., 10-20 min/day) and in 5 it was not  – The stimulation frequency varied between 1-10 Hz – In most studies the intensity of stimulation was set based on the RMT of the mylohyoid / tongue muscle – The site of stimulation was the ipsilesional/contralesional mylohyoid cortical area / pharyngeal motor cortical area / esophageal cortical area / cerebellum (1 study) | – Variation  across studies (6 studies used more than one tool)  – Clinical and/or instrumental evaluation (VFS, FEES) | – Limited, up to 2 months) | – Significant improvement immediately, at 1 month and 2 months post rTMS compared to standard physical-medical treatment – No statistically significant difference between subgroups according to stimulation site | – Immediate effects  0.85 [0.45, -1.24]  – At 1 month post-treatment  0.88 [0.41, -1.35]  – At 2 months post-treatment  2.28 [0.28, -4.27]  – Stimulation ipsilesionally 1.13 [0.53, -1.73]  – Stimulation contralesionally 0.78 [0.49, -1.07]  – Stimulation bilaterally 1.18 [0.59, -1.76] | – Natural Science  Foundation of Zheijiang Province (LY21H170001) |
| 6. Qiao et al. (2022) with meta-analysis | – 12 studies with 433 participants in total | – Chronicity measured in days (8-756)  – Hemorrhage / infarction | – The duration of treatment ranged from 1 to 10 days – The duration of a single stimulation session varied between 5-20 min   – The stimulation frequency ranged from 1 to 10 Hz – The site of stimulation was the cerebellum or the affected/unaffected hemisphere – The ST ranged from 42-110% | – The primary studies employed various tools (clinical and instrumental), but the SR prioritized DG and PAS to measure dysphagia severity | – Not reported | – Significant advantage of rTMS interventions compared to control conditions.  – Treatment duration: treatment >5 days showed a higher effect size, but the result in the <5-day group was opposite.  – Stroke chronicity: rTMS in the subacute phase (<60 days) showed significantly greater benefits than control conditions, but rTMS at the recovery phase (>60 days) did not outperform control  – Stimulation duration: studies with ≤10 min and studies with >10 min showed a higher effect size than the control conditions – Stimulation frequency: studies with low and high frequency led to a higher effect size than the control conditions – Stimulation location: stimulation of the affected and the unaffected hemisphere had higher effect sizes than control conditions – Stimulation pattern: the rTMS group showed significant effect sizes compared to the control condition with high frequency in the affected hemisphere, high frequency in the unaffected hemisphere and low frequency in the unaffected hemisphere  – In the age subgroup analysis, significant effect sizes were found for all age groups compared to control condition  – Sensitivity analyses excluding low-quality studies: lower effect size when treatment duration was less than 5 days and significantly higher effect size in the low-frequency group | – rTMS vs control: -0.67 [-0.88, -0.45] − Treatment duration: >5 days: -0.80 [-1.08, -0.52] <5 days: -0.50 [-1.26, 0.26] – Stroke chronicity:  rTMS in the subacute phase: -0.60 [-0.85, -0.35] rTMS at the recovery phase: -0.32 [-0.72, 0.08]  – Stimulation duration: ≤10 min -0.71 [-1.02, -0.40] >10 min -0.64 [-0.93, -0.36] – Stimulation frequency: low frequency -1.01 [-1.64, -0.38] high frequency -0.58 [-0.91, -0.25] – Stimulation location: affected hemisphere -0.73 [-1.21, -0.26] unaffected hemiesphere -1.07 [-1.45, -0.69] – Stimulation pattern:  high frequency in the affected hemisphere -0.88 [-1.51, -0.24] high frequency in the unaffected hemiesphere  -0.59 [-1.07, -0.1] low frequency in the unaffected hemisphere -1.01 [-1.64, -0.38] – Age: ≥65 years -0.41 [-0.75, -0.4] <65 years -0.93 [-1.4, -0.46] – Sensitivity analysis: duration <5 days -0.11 [-0.57, 0.34] low frequency -1.28 [-1.81, -0.76] | – Natural Science Foundation of China (NSFC, No. 81972159) and Natural Science Foundation of Guangdong Province (NSFP, No. 2020A1515010881). |
| 7. Tan et al. (2022) with meta-analysis | – 9 studies with 238 participants in total | – Subacute and chronic  – Hemorrhage / infarction | – TMS applied as a standalone vs an adjuvant therapy – The duration of treatment ranged from 5 to 10 days – The number of pulses varied between 300-3000  – The stimulation frequency ranged from 1 to 10 Hz – The site of stimulation was the mylohyoid cortical area, the tongue area of the motor cortex, the esophageal cortical area or the pharyngeal motor cortex of the affected / unaffected hemisphere or of both hemispheres – The RMT ranged from 42-110% | – The primary studies employed various tools (clinical and instrumental) | – 5 days to 6 months | – Large effect of rTMS in improving swallowing function – Large heterogeneity observed, however, subgroup analyses were conducted across all NIBS studies – The effect of rTMS on swallowing function during follow-up was not significant | – rTMS vs control: *g =* -0.86 [-1.57, -0.16] – rTMS vs control (follow-up): *g =* -0.90 [-1.88, 0.09] | – No financial support |
| 8. Wen et al. (2022) with meta-analysis | – 11 studies with 463 participants in total | – Subacute and chronic measured in months  – Hemorrhage / infarction | – TMS applied as a standalone vs an adjuvant therapy – The duration of treatment ranged from 5-14 days – All studies but one applied therapy once a day and one study twice a day – The duration of each session varied between 10 and 20 min  – The number of pulses varied between 250 and 2400  – The stimulation frequency ranged from 1 to 10 Hz – The site of stimulation was the mylohyoid cortical area, the esophageal cortical area or the pharyngeal motor cortex of the affected / unaffected hemisphere or of both hemispheres – The RMT ranged from 90-130% | – The primary studies employed various tools (clinical and/or instrumental) | – Not reported | – Large effect of rTMS group compared to control group – No differences between traditional therapy only control groups and traditional therapy with sham rTMS control groups – No differences between affected, unaffected, or bilateral stimulation protocols – Both HF and LF protocols were effective, but HF effects were significantly greater – No differences were found between 1, 3, or 5 Hz protocols | – rTMS vs control: 2.15 [1.61, 2.70] – rTMS vs control (traditional therapy): 2.03 [1.26, 2.80] – rTMS vs control (traditional therapy and sham): 2.23 [1.49, 2.97] – rTMS vs control (affected hemisphere): 1.99 [1.11, 2.88] – rTMS vs control (unaffected hemisphere): 1.99 [1.34, 2.64] – rTMS vs control (bilatteral stimulation): 4.07 [-1.09, 9.23] – rTMS vs control (HF): 2.50 [1.84, 3.16] – rTMS vs control (LF): 1.26 [0.61, 1.90] – rTMS vs control (5 Hz): 2.03 [1.26, 2.81]  – rTMS vs control (3Hz): 2.28 [1.53, 3.04]  –rTMS vs control (1Hz): 1.26 [1.40, 1.90] | – The authors declare that the research was conducted in the absence of any commercial or financial relationships that could be construed as a potential conflict of interest |
| 9. Xie et al. (2022) with meta-analysis | – 10 studies with 246 participants in total | – Chronicity not reported  – Hemorrhage / infarction | – TMS applied as a standalone vs an adjuvant therapy – The duration of treatment was reported in the number of times and ranged from 1 to 10 – The duration of each session was reported in min or number of pulses and ranged from 10 to 20 min and 250 to 1200 pulses, respectively  – The stimulation frequency ranged from 1 to 10 Hz – The site of stimulation was the mylohyoid cortical area, the esophageal cortical area or the pharyngeal motor/sensory cortex of the affected / unaffected hemisphere or of both hemispheres – The RMT ranged from 90-130% | – The primary studies employed various tools (clinical and/or instrumental) – Separate meta-analyses for each outcome: (1) DD; (2) FDS; (3) VDS; (4) PAS; (5) BI; (6) dropout rate; (7) adverse effects | – Not reported | – Overall swallowing function:  rTMS significantly improved overall swallowing ability  – Subgroup Analysis of Overall Swallowing Function:  According to stimulation site:  bilateral hemispheric stimulation may lead to better overall swallowing gains in comparison to stimulation of the ipsilesional / contralesional hemisphere According to stimulation frequency: LF-rTMS treatment produced better effects on overall swallowing function than HF-rTMS treatment  – PAS: rTMS significantly reduced accidental aspiration compared to controls – Subgroup Analysis of PAS According to stimulation site:  bilateral stimulation may produce better therapeutic effects on overall swallowing function in comparison to ipsilesional / contralesional stimulation  According to stimulation frequency:  LF-rTMS treatment produced better effects on overall swallowing function than HF- rTMS treatment   – BI:  rTMS significantly improved activity of daily living compared to the control group  Dropout Rate:  No differences in dropout rate between the rTMS group and the control group  Adverse Effects: No differences in adverse effects between the rTMS group and the control group | – Overall swallowing function:  -0.76 [-1.07, -0.46]  – Subgroup Analysis of Overall Swallowing Function:  According to stimulation site:  bilateral hemispheric stimulation −1.15 [−1.87, −0.43]  ipsilesional hemispheric stimulation  −0.74 [−1.69, 0.20]  contralesional hemispheric stimulation  −0.59 [−1.14, −0.05]  According to stimulation frequency  HF-rTMS: −0.70 [−1.33, −0.06] LF-rTMS: −0.86 [−1.16, −0.34]  – PAS: −1.03 [−1.51, −0.55]  – Subgroup Analysis of PAS According to stimulation site:  Ipsilesional hemispheric stimulation −0.09 [−0.95, 0.78]  Contralesional hemispheric stimulation  −1.37 [−2.00, −0.75]  Bilateral hemispheric stimulation  −2.06 [−3.71, −0.41] According to stimulation frequency:  HF-rTMS: −0.60 [−1.31, −0.10] LF-rTMS −1.42 [−2.09, −0.75]  – BI:  23.86 [12.73, 34.99]  Dropout Rate  RR 0.87 [0.38, 2.00] Adverse Effects  RR 2.61 [0.69, 9.86] | - Sichuan Medical Research Project Plan [Q18038]; Research and Development Project of Affiliated Hospital of North Sichuan Medical College [2021ZD014] and China Nanchong City-School Cooperative Scientific Research Special Fund [19SXHZ0103] |
| 10. Zhu & Gu (2022) with meta-analysis | – 7 studies with 186 participants in total | – Acute, subacute, chronic provided as mean and sd for days / weeks / months – No details regarding the type of stroke – One study did not provide any details (i.e., Khedr et al., 2009) | – rTMS 1, 3, 5, or 10 Hz – 5 days to 2 weeks therapy duration – Variety of stimulation targets (i.e., motor cortex, tongue area of the motor cortex, swallowing motor cortex, pharyngeal motor cortex, anterolateral scalp) including ipsilesional, contralesional, and bilateral stimulation for 300 to 3000 pulses per session at 90, 100, 120% or N/A % of RMT | – Variations across studies (DSRS, FEDSS, FOIS, PAS, VDS)  – In some studies, >1 scale was conducted to evaluate swallowing function | – Various follow ups with up to 12 months for one study | – Increase of swallowing funtion favoring rTMS treatment compared to sham.  – Subgroup analyses (Asians/Caucasians) indicate significant rTMS benefits for Asians (insignificant for caucasians but with k = 3 and high error) – Similar effect of high-frequency rTMS and low-frequency rTMS but the authors only report: "Subgroup analyses show an increase in the swallowing function in poststroke dysphagia given high-frequency (HF) rTMS, compared to patients treated with sham rTMS" with no mention on low-frequency results | – rTMS vs control 1.08 [0.37, 1.80] – rTMS vs control Asian 0.83 [0.36, 1.29] – rTMS vs control Caucasian 2.69 [-1.12, 6.49] – High-frequency rTMS vs control 1.14 [0.24, 2.03] – Low-frequency rTMS vs control 1.04 [0.24, 1.72] | – National Natural Science Foundation of China (No. 81901108; No. 82001125), Natural Science Foundation of Ji- angsu Province (No. BK20180201), the Foundation for Jiangsu Entrepreneurship and Innovation Doctors. |
| 11. Cheng et al. (2021) with meta-analysis | – 13 studies with 335 participants in total | – Variety of time post stroke, up to 6 years  – Ischemic, haemorrhagic or brainstem infarction | – rTMS 1, 3, 5, or 10 Hz  – Ipsilisional, contralisional, or bilateral stimulation on esophageal, mylohoid, tongue, pharyngeal motor cortex, or pharyngeal sensory cortex at 90, 100, 120, or 130% of rMT – Stimulation time varied from 2-20 minutes and therapy duration was between 1 to 10 days | – Variations across studies (DD, MASA, PAS, PTT, SSA) | – Various follow ups with up to 12 months for one study | – Overall moderate effect of rTMS  – Moderate effect for early and intermediate follow-up, but no significant effect for late follow-up  – High frequency rTMS over the ipsilesional hemisphere showed larger positive effects  – Low-frequency ipsilesional or contralesional rTMS effects are comparable but not as large as high-frequency ipsilesional  – The stronger effect of high-frequency ipsilesional could be due to the effects of one specific study, which once removed, the effect becomes non-significant (Khedr 2009) | – rTMS vs control  0.73 [0.49, 0.98] – rTMS vs control early follow-up 0.69 [0.46, 0.93] – rTMS vs control intermediate follow-up 1.02 [0.45, 1.69]  – rTMS vs control late follow-up 0.78 [-0.08, 1.65]  – rTMS vs control low-frequency contralesional 0.61 [0.23, 0.98]  – rTMS vs control high-frequency ipsilesional 0.83 [0.14, 1.52]  – rTMS vs control high-frequency contralesional 0.59 [0.03, 1.14] | – MRC, Wellcome Trust, Stroke Association, and NIHR |
| 12. Li et al. (2021) with meta-analysis | – 11 studies with 507 participants in total | – Acute, subacute, chronic, provided as mean and sd for hours / days /weeks / months – No details regarding the type of stroke – One study did not provide any details (i.e., Fang et al., 2020) | – rTMS 1, 3, 5, or 10 Hz – 1 to 4 weeks therapy duration – Variety of stimulation targets (i.e., pharyngeal motor cortex; from the anterolateral cortex of the skull to the front of the primary motor area, the lowest part of the anterior central gyrus, and the back part of the inferior frontal gyrus; suprahyoid brain area) including ipsilesional, contralesional, and bilateral stimulation | – Variations across studies (DOSS, FDS, PAS, SSA, WST)  – Meta-analyses were conducted seperately for each outcome | – Not reported | – All meta-analyses for each independent outcome indicated a significant large effect of rTMS therapy compared to control | – rTMS vs control on DOSS 2.58 [2.04, 3.12] – rTMS vs control on SSA -1.29 [-1.83, -0.75] – rTMS vs control on PAS -0.90 [-1.43, -0.37] – rTMS vs control on FDS -1.33 [-1.91, -0.75] – rTMS vs control on WST 6.23 [5.44, 7.03] | - No information provided |
| 13. Wang et al. (2021) with meta-analysis | – 8 studies with 249 participants in total | – Acute, recovery, or sequelae phase with no specific timings clarified – Ischemia / hemorrhage | – rTMS 1, 3, 5, or 10 Hz  – 5 or 10 days therapy duration  – 300, 500, 600, or 1200 pulses (5 to 20 minutes of stimulation)  – Ipsilesional, contralesional, or bilatteral stimulation, on esophageal, mylohyoid, pharyngal hot spot at 90, 100, 110, 120, or 130% MT | – Variations across studies (DG, PAS, SSA) | – Not reported | – Large significant effect of rTMS compared to control | – rTMS vs control: 1.27 [0.59, 1.95] | – Research & Development Projects of Shandong Province (Grant No. 2019GSF108262); Shandong Provincial Natural Science Foundation |
| 14. Yang et al. (2021) with meta-analysis | – 7 studies with 186 participants in total | – No details regarding stroke chronicity – Ischemic / hemorrhagic stroke | – rTMS 1, 3, 5, or 10 Hz  – Ipsilesional, contralesional, or bilateral stimulation on mylohyoid, esophagus, or pharyngeal area at 90, 100, 120 or 130% MT – No data on stimulation time or therapy duration reported | – Variations across studies (DG, DOSS, FDS, PAS, PTT, VDS) | – Not reported | – Significant effect of rTMS on swalling function compared to control group – Subgroup analyses based on rTMS frequency (low vs. high) indicated a similar effect; however, it was not significant for either group | – rTMS vs control 0.65 [0.04, 1.26]  – Low-frequency rTMS vs control 0.68 [-0.28, 1.63]  – High-frequency rTMS vs control 0.69 [-0.15, 1.53] | – 2020 District Level Scientific Research Project of Longhua District Medical (ID:2020040) and Health Institutions and Shenzhen Longhua District Rehabilitation Medical Equipment Development and Transformation Joint Key Laboratory |
| 15. Bath et al. (2018) with meta-analysis | – 6 studies with 167 participants in total | – Acute or subacute (i.e., within 6 months of onset)  – Ischemic / hemmorhagic | – rTMS protocols of 1, 3, or 5 Hz  – Ipsilesional or contralesional stimulation | – Variations across studies  – The authors focused on swallowing ability, case fatality at the end of trial, and PAS | – Not reported | – The authors report that at the end of trial TMS improved swallowing ability, however, the finding could be due to chance, considering no subgroup differences and heterogeneity – No effects of TMS were found on PAS or case fatality at the end of trial. | – rTMS vs control case fatality after trial: 0.28 [0.03, 2.93]  – rTMS vs control Swallowing ability: 1.29 [0.21, 2.37]  – rTMS vs control PAS: 0.53 [0.16, 1.22] | – No information provided |
| 16. Chiang et al. (2018) with meta-analysis | – 6 studies with  170 participants in total | – Within 3 months of onset  – Ischemic / hemmorhagic | – rTMS protocols 1, 3, or 5 Hz – Stimulation intensity at 90, 100, 120, or 130% of RMT – Site of stimulation was the affected or bilateral esophageal motor cortex, unaffected pharyngeal hot spot, affected or unaffected hemipshere, and bilateral, or unilateral mylohyoid hotspot | – Variations across studies (DG, PAS, or SSA) | – Limited, up to 2 weeks | – Large effect of rTMS | – rTMS vs control 1.06 [0.64, 1.47] | – No information provided |
| 17. Liao et al. (2017) with meta analysis | – 6 studies with 163 participants in total | – Scute & sub-acute  (within 3 months post-stroke)  – Ischemic / hemmorhagic | – rTMS protocols 1, 3, or 5 Hz at 90, 100, 120, or 130% of RMT / MT – Stimulated area: affected, unaffected, or both hemispheres' esophageal cortex – Duration of therapy was either 1 or 2 weeks, with a number of total daily pulses varying (300, 500, 1000, or 1200) | – Variation across studies (DG, PAS, or SSA) | – Limited, up to 3 months | – Large effect of rTMS – Higher effects of rTMS for high-frequency protocols compared to low-frequency protocols – Stimulation ineffective in affected hemisphere; effective in unaffected or bilateral | – rTMS vs control 1.24 [0.67, 1.81]  – rTMS vs control low-frequency 1.02 [0.51, 1.53]  – rTMS vs control high-frequency 1.38 [0.47, 2.29]  – rTMS vs control unnaffected hemisphere 0.91 [0.48, 1.35]  – rTMS vs control affected hemisphere 1.59 [-0.14, 3.31]  – rTMS vs control bilateral 1.60 [0.57, 2.63]  – rTMS vs control follow-up 1.91 [0.92, 2.90] | – Natural Science Foundation of China [NSFC, No. 81271559], the Basic Program Funded by Science & Technology Department of Sichuan Province [No. 12JC0565] and the State Administration of Foreign Experts Affairs, the P.R. of China [No. 20125100024] |
| 18. Pisegna et al. (2016) wih meta-analysis | – 4 studies with 56 participants in total | – Acute, subacute and chronic (up to 40 months post-stroke) – Unilateral ischemic/hemorrhagic stroke | – rTMS protocols 1, 3, or 5 HZ at 90, 100, or 120% of RMT – Stimulated area: affected, unaffected pharyngeal, mylohyoid, or esophageal motor cortex, with the exception of one study which stimulated both hemispheres depending on the protocol – Protocol varied across the studies in terms of treatment duration (1-10 days), duration of stimulation (10 min/day with one study not reporting data), and number of pulses (between 1 block of 1200 pulses and 20 blocks of 50 pulses) | – Variation across studies, but the authors reported the use of PAS in 3 studies, FDS in 2 studies, and the Awareness of Dysphagia Scale in one study, which the authors used for their analyses. | – Only one rTMS study reported follow-up results at 2 weeks | – Overall moderate effect of rTMS | – rTMS vs control 0.56 [0.04, 1.09] – rTMS vs control when excluding the study of Khedr et al. (2009): 0.44 | – Partial funding from National Institutes of Health (NIDCD 1R01DC012584-01A1) and National Institute On Deafness And Other Communication Disorders of the National Institutes of Health (R01DC012584) |
| 19. Yang et al. (2015) with meta-analysis | – 3 studies with 66 participants in total | – Acute & subacute (1-114 days post-stroke) – No details on type of stroke (ischemic vs hemmorhagic) | – rTMS protocols 3-5 Hz – 10-minute sessions for 5 or 10 days with 10 stimulations per minute – Stimulated area: affected, unaffected, or both hemispheres' esophageal / pharyngeal cortex | – The autors report instrumental evaluation as the main outcome of the included studies (DOSS for 2 studies, and VFS for 1 study) | – Limited follow up for 2 of the studies (up to 2 months) | – Large effect of rTMS | – rTMS vs control 1.61 [0.59, 2.63] | – No information provided |
| Key: BI: Barthel index scale; CI: confidence interval; CT: computed tomography; DD: Degree of Dysphagia; DG: Dysphagia Grade; DOSS: Dysphagia Outcome Severity Scale; DSRS: Dysphagia Severity Rating Scale; FDS: Functional Dysphagia Scale; FEDSS: Fiberoptic Endoscopic Dysphagia Severity Scale; FEES: Fiberoptic Endoscopic Examination of Swallowing; FOIS: Functional Oral Intake Scale; HF: High-Frequency; IPES: Intrapharyngeal Electrical Stimulation; LF: Low-Frequency; MASA: Mann Assessment of Swallowing Ability; min: minutes; MRI: Magnetic Resonance Imaging; MT: Motor Threshold; N/A: Non-Applicable; NIBS: Non-Invasive Brain Stimulation; NMES: Neuromuscular Electrical Stimulation; OTT: Oral Transit Time; PAS: Penetration - Aspiration Scale; PTT: Pharyngeal Transit Time; RMT: Resting Motor Threshold; RR: Risk-Ratio; rTMS: repetitive Transcranial Magnetic Stimulation; SMD: Standardized Mean Difference; ST: Stimulation Threshold; SR: Systematic Review; SSA: Standardized Swallowing Assessment; TBI: Traumatic Brain Injury; TDT: Traditional Dysphagia Therapy; VDS: Videofluoroscopic Dysphagia Scale; VFS: Videofluoroscopy; WST: Water Swallow Test | | | | | | | | |

**Supplementary Material 3.** Overview of the characteristics of the included systematic reviews with/without meta-analyses exploring the utilization of TMS in post-stroke dysphagia rehabilitation
