# Supplementary material for: The effectiveness of transcranial magnetic stimulation for dysphagia in stroke patients: an umbrella review of systematic reviews and meta-analyses
Source: Front Hum Neurosci. 2024 Mar 14;18:1355407. doi: 10.3389/fnhum.2024.1355407 (PMC10972992; doi:10.3389/fnhum.2024.1355407)
Supplement: Supplementary file 5 [file Data_Sheet_5.docx]

| **Review** | **Study** | **TMS_m** | **TMS_sd** | **TMS_n** | **CTR_m** | **CTR_sd** | **CTR_n** | **Total_n** |  | |
| --- | --- | --- | --- | --- | --- | --- | --- | --- | --- | --- |
| 3. Banda et al., (2023) | Cabib 2020 |  |  |  |  |  |  | 30 |  | |
| 6. Qiao et al., (2022) | Cabib 2020 | -0.7 | 2.29 | 12 | -0.5 | 2.3 | 12 | 24 |  | |
| 14. Cheng et al. (2021) | Cabib 2020 |  |  | unclear |  |  | 12 | unclear |  | |
| 11. Li et al. (2021) | Cai 2019 |  |  | 20 |  |  | 20 | 40 |  | |
| 8. Wen et al., (2022) | Cai 2019a | 2.65 | 0.27 | 20 | 0.65 | 0.32 | 10 | 30 |  | |
| 8. Wen et al., (2022) | Cai 2019b | 1.6 | 0.24 | 20 | 0.65 | 0.32 | 10 | 30 |  | |
| 3. Banda et al., (2023) | Cheng 2017 |  |  |  |  |  |  | 28 |  | |
| 10. Zhu & Gu (2022) | Cheng 2017 |  |  | 11 |  |  | 4 | 15 |  | |
| 14. Cheng et al. (2021) | Cheng 2017 |  |  | 11 |  |  | 4 | 15 |  | |
| 1. Balcerak et al. (2022) | Du 2016 |  |  |  |  |  |  | 40 |  | |
| 4. Hsiao et al., (2023) | Du 2016 | -2.14 | 2.49 | 15 | -0.29 | 2.07 | 12 | 27 |  | |
| 5. Li et al., (2022) | Du 2016 | 1.44 | 2.24 | 28 | 0.08 | 2.04 | 12 | 40 |  | |
| 6. Qiao et al., (2022) | Du 2016 | -2 | 1.4 | 28 | 0 | 0.79 | 12 | 40 |  | |
| 10. Zhu & Gu (2022) | Du 2016 |  |  | unclear |  |  | 12 | unclear |  | |
| 16. Chiang et al. (2018) | Du 2016 |  |  | Unclear |  |  | Unclear | Unclear |  | |
| 4. Hsiao et al., (2023) | Du 2016 | -1.34 | 1.27 | 13 | -0.29 | 1.44 | 12 | 25 |  | |
| 7. Tan et al., (2022) | Du 2016a | 3.17 | 1.11 | 13 | 4.08 | 1.3 | 6 | 19 |  | |
| 8. Wen et al., (2022) | Du 2016a | 0.18 | 0.13 | 13 | 0.1 | 0.34 | 6 | 19 |  | |
| 9. Xie et al., (2022) | Du 2016a | 2.64 | 0.83 |  | 3.37 | 0.84 | 6 | 6 |  | |
| 12. Wang et al. (2021) | Du 2016a |  |  | 15 |  |  | 12 | 27 |  | |
| 14. Cheng et al. (2021) | Du 2016a |  |  | 13 |  |  | 12 | 25 |  | |
| 15. Bath et al. (2018) | Du 2016a |  |  | 13 |  |  | 6 | 19 |  | |
| 17. Liao et al. (2017) | Du 2016a |  |  | 26 |  |  | 12 | 38 |  | |
| 7. Tan et al., (2022) | Du 2016b | 3.33 | 0.74 | 13 | 4.08 | 1.3 | 6 | 19 |  | |
| 8. Wen et al., (2022) | Du 2016b | 0.72 | 0.3 | 15 | 0.1 | 0.13 | 6 | 21 |  | |
| 9. Xie et al., (2022) | Du 2016b | 2.37 | 0.84 | 13 | 3.37 | 0.84 | 6 | 19 |  | |
| 12. Wang et al. (2021) | Du 2016b |  |  | 13 |  |  | 12 | 25 |  | |
| 14. Cheng et al. (2021) | Du 2016b |  |  | 13 |  |  | 12 | 25 |  | |
| 15. Bath et al. (2018) | Du 2016b |  |  | 13 |  |  | 6 | 19 |  | |
| 17. Liao et al. (2017) | Du 2016b |  |  | 26 |  |  | 12 | 38 |  | |
| 11. Li et al. (2021) | Fang 2020 |  |  | 30 |  |  | 30 | 60 |  | |
| 11. Li et al. (2021) | Jiao 2019 |  |  | 30 |  |  | 30 | 60 |  | |
| 11. Li et al. (2021) | Jiao 2020 |  |  | 20 |  |  | 20 | 40 |  | |
| 1. Balcerak et al. (2022) | Khedr 2009 |  |  |  |  |  |  | 26 |  | |
| 5. Li et al., (2022) | Khedr 2009 | 2.07 | 0.6 | 14 | 0.29 | 0.72 | 12 | 26 |  | |
| 6. Qiao et al., (2022) | Khedr 2009 | -2.2 | 0.81 | 14 | -0.5 | 1.03 | 12 | 26 |  | |
| 8. Wen et al., (2022) | Khedr 2009 | 2.07 | 0.6 | 12 | 0.29 | 0.72 | 12 | 24 |  | |
| 9. Xie et al., (2022) | Khedr 2009 | 1.36 | 1.24 | 14 | 3.43 | 0.73 | 11 | 25 |  | |
| 10. Zhu & Gu (2022) | Khedr 2009 |  |  | 14 |  |  | 12 | 26 |  | |
| 12. Wang et al. (2021) | Khedr 2009 |  |  | 14 |  |  | 11 | 25 |  | |
| 13. Yang et al. (2021) | Khedr 2009 |  |  | 14 |  |  | 12 | 26 |  | |
| 14. Cheng et al. (2021) | Khedr 2009 |  |  | 14 |  |  | 12 | 26 |  | |
| 15. Bath et al. (2018) | Khedr 2009 |  |  | 14 |  |  | 12 | 26 |  | |
| 16. Chiang et al. (2018) | Khedr 2009 |  |  | 14 |  |  | 12 | 26 |  | |
| 17. Liao et al. (2017) | Khedr 2009 |  |  | 14 |  |  | 12 | 26 |  | |
| 18.Pisegna et al. (2016) | Khedr 2009 | 2.05 | 1.26 | 14 | 0.28 | 1.73 | 12 | 26 |  | |
| 19. Yang et al. (2015) | Khedr 2009 | 2.07 | 0.6 | 14 | 0.29 | 0.72 | 12 | 26 |  | |
| 1. Balcerak et al. (2022) | Khedr 2010 |  |  |  |  |  |  | 22 |  | |
| 6. Qiao et al., (2022) | Khedr 2010 | -0.9 | 1.9 | 11 | -0.8 | 1.83 | 11 | 22 |  | |
| 7. Tan et al., (2022) | Khedr 2010 | 1.4 | 0.4 | 11 | 3.7 | 0.5 | 11 | 22 |  | |
| 8. Wen et al., (2022) | Khedr 2010 | 1.7 | 1.12 | 11 | 0.03 | 1 | 11 | 22 |  | |
| 9. Xie et al., (2022) | Khedr 2010 | 1.76 | 1.61 | 11 | 3.8 | 1.26 | 11 | 22 |  | |
| 10. Zhu & Gu (2022) | Khedr 2010 |  |  | unclear |  |  | unclear | unclear |  | |
| 15. Bath et al. (2018) | Khedr 2010 |  |  | 11 |  |  | 11 | 22 |  | |
| 19. Yang et al. (2015) | Khedr 2010 | 1.7 | 1.12 | 11 | 0.03 | 1 | 11 | 22 |  | |
| 12. Wang et al. (2021) | Khedr 2010a |  |  | 6 |  |  | 5 | 11 |  | |
| 13. Yang et al. (2021) | Khedr 2010a |  |  | 6 |  |  | 5 | 11 |  | |
| 14. Cheng et al. (2021) | Khedr 2010a |  |  | 5 |  |  | 6 | 11 |  | |
| 16. Chiang et al. (2018) | Khedr 2010a |  |  | 6 |  |  | 5 | 11 |  | |
| 17. Liao et al. (2017) | Khedr 2010a |  |  | 11 |  |  | 11 | 22 |  | |
| 12. Wang et al. (2021) | Khedr 2010b |  |  | 5 |  |  | 6 | 11 |  | |
| 13. Yang et al. (2021) | Khedr 2010b |  |  | 5 |  |  | 6 | 11 |  | |
| 14. Cheng et al. (2021) | Khedr 2010b |  |  | 6 |  |  | 5 | 11 |  | |
| 16. Chiang et al. (2018) | Khedr 2010b |  |  | 5 |  |  | 6 | 11 |  | |
| 17. Liao et al. (2017) | Khedr 2010b |  |  | 11 |  |  | 11 | 22 |  | |
| 6. Qiao et al., (2022) | Kim 2011 | -1.8 | 2.3 | 20 | -0.7 | 1.2 | 10 | 30 |  | |
| 10. Zhu & Gu (2022) | Kim 2011 |  |  | unclear |  |  | 10 |  |  | |
| 4. Hsiao et al., (2023) | Kim 2011 | 0.6 | 1 | 10 | 0.7 | 1.2 | 20 | 30 |  | |
| 4. Hsiao et al., (2023) | Kim 2011 | -3 | 2.6 | 10 | -0.7 | 1.2 | 10 | 20 |  | |
| 8. Wen et al., (2022) | Kim 2011a | 3 | 2.6 | 10 | 0.7 | 1.2 | 5 | 15 |  | |
| 9. Xie et al., (2022) | Kim 2011a | 9.09 | 2.67 | 10 | 11.08 | 4.55 | 5 | 15 |  | |
| 13. Yang et al. (2021) | Kim 2011a |  |  | 10 |  |  | 10 | 20 |  | |
| 14. Cheng et al. (2021) | Kim 2011a |  |  | 10 |  |  | 10 | 20 |  | |
| 18.Pisegna et al. (2016) | Kim 2011A | 0.6 | 1 | 10 | 0.7 | 1.2 | 10 | 20 |  | |
| 8. Wen et al., (2022) | Kim 2011b | 0.6 | 1 | 10 | 0.7 | 1.2 | 5 | 15 |  | |
| 9. Xie et al., (2022) | Kim 2011b | 8.47 | 3.23 | 10 | 11.08 | 4.55 | 5 | 15 |  | |
| 13. Yang et al. (2021) | Kim 2011b |  |  | 10 |  |  | 10 | 20 |  | |
| 14. Cheng et al. (2021) | Kim 2011b |  |  | 10 |  |  | 10 | 20 |  | |
| 18.Pisegna et al. (2016) | Kim 2011B | 3 | 2.6 | 10 | 0.7 | 1.2 | 10 | 20 |  | |
| 15. Bath et al. (2018) | Kim 2012a |  |  | 10 |  |  | 10 | 20 |  | |
| 15. Bath et al. (2018) | Kim 2012b |  |  | 10 |  |  | 5 | 15 |  | |
| 17. Liao et al. (2017) | Kim 2013a |  |  | 20 |  |  | 10 | 30 |  | |
| 17. Liao et al. (2017) | Kim 2013b |  |  | 20 |  |  | 10 | 30 |  | |
| 8. Wen et al., (2022) | Li 2021a | 5.5 | 2.37 | 12 | 1.1 | 2.51 | 4 | 16 |  | |
| 8. Wen et al., (2022) | Li 2021b | 9.5 | 2.14 | 12 | 1.1 | 2.51 | 4 | 16 |  | |
| 8. Wen et al., (2022) | Li 2021c | 8.5 | 2.3 | 13 | 1.1 | 2.51 | 4 | 17 |  | |
| 1. Balcerak et al. (2022) | Lim 2014 |  |  |  |  |  |  | 29 |  | |
| 3. Banda et al., (2023) | Lim 2014 |  |  |  |  |  |  | 60 |  | |
| 5. Li et al., (2022) | Lim 2014 | 14.4 | 5.61 | 20 | 7.95 | 4.68 | 20 | 40 |  | |
| 6. Qiao et al., (2022) | Lim 2014 | -2.08 | 1.07 | 15 | -0.92 | 0.76 | 14 | 29 |  | |
| 8. Wen et al., (2022) | Lim 2014 | 2.08 | 1.07 | 15 | 0.92 | 0.76 | 14 | 29 |  | |
| 9. Xie et al., (2022) | Lim 2014 | 32.65 | 11.18 | 14 | 46.3 | 13.8 | 15 | 29 |  | |
| 11. Li et al. (2021) | Lim 2014 |  |  | 14 |  |  | 15 | 29 |  | |
| 12. Wang et al. (2021) | Lim 2014 |  |  | 14 |  |  | 15 | 29 |  | |
| 13. Yang et al. (2021) | Lim 2014 |  |  | 14 |  |  | 15 | 29 |  | |
| 14. Cheng et al. (2021) | Lim 2014 |  |  | unclear |  |  | 15 | unclear |  | |
| 16. Chiang et al. (2018) | Lim 2014 |  |  | 14 |  |  | 15 | 29 |  | |
| 17. Liao et al. (2017) | Lim 2014 |  |  | 14 |  |  | 15 | 29 |  | |
| 4. Hsiao et al., (2023) | Lim 2014 | -2.54 | 1.34 | 14 | -2 | 1 | 15 | 29 |  | |
| 6. Qiao et al., (2022) | Lin 2018 | -1.2 | 2.2 | 13 | 0 | 2.3 | 15 | 28 |  | |
| 12. Wang et al. (2021) | Lin 2018 |  |  | 13 |  |  | 15 | 28 |  | |
| 14. Cheng et al. (2021) | Michou 2014 |  |  | 6 |  |  | 6 | 12 |  | |
| 18.Pisegna et al. (2016) | Michou 2014 | 0.21 | 2.49 | 6 | 0.3 | 2.38 | 6 | 12 |  | |
| 11. Li et al. (2021) | Ou 2019 |  |  | 20 |  |  | 20 | 40 |  | |
| 8. Wen et al., (2022) | Ouyang 2019 | 2.7 | 0.55 | 20 | 1.85 | 0.44 | 20 | 40 |  | |
| 3. Banda et al., (2023) | Park 2013 |  |  | 9 |  |  | 9 | 18 |  | |
| 5. Li et al., (2022) | Park 2013 | 8.37 | 8.04 | 9 | 2.2 | 7.05 | 9 | 18 |  | |
| 6. Qiao et al., (2022) | Park 2013 | -1.48 | 2.04 | 9 | -0.3 | 2.1 | 9 | 18 |  | |
| 7. Tan et al., (2022) | Park 2013 | 1.93 | 1.52 | 9 | 3 | 2.17 | 9 | 18 |  | |
| 8. Wen et al., (2022) | Park 2013 | 1.47 | 0.62 | 9 | 0.3 | 0.66 | 9 | 18 |  | |
| 9. Xie et al., (2022) | Park 2013 | 25.3 | 9.8 | 9 | 21.2 | 15.6 | 9 | 18 |  | |
| 10. Zhu & Gu (2022) | Park 2013 |  |  | 9 |  |  | 9 | 18 |  | |
| 11. Li et al. (2021) | Park 2013 |  |  | 9 |  |  | 9 | 18 |  | |
| 12. Wang et al. (2021) | Park 2013 |  |  | 12 |  |  | 12 | 24 |  | |
| 13. Yang et al. (2021) | Park 2013 |  |  | 9 |  |  | 9 | 18 |  | |
| 14. Cheng et al. (2021) | Park 2013 |  |  | 9 |  |  | 9 | 18 |  | |
| 15. Bath et al. (2018) | Park 2013 |  |  | not reported |  |  | not reported | not reported |  | |
| 16. Chiang et al. (2018) | Park 2013 |  |  | 9 |  |  | 9 | 18 |  | |
| 17. Liao et al. (2017) | Park 2013 |  |  | 9 |  |  | 9 | 18 |  | |
| 18.Pisegna et al. (2016) | Park 2013 | 1.48 | 1.96 | 9 | 0.3 | 2.1 | 9 | 18 |  | |
| 19. Yang et al. (2015) | Park 2013 | 8.37 | 8.04 | 9 | 2.2 | 7.05 | 9 | 18 |  | |
| 3. Banda et al., (2023) | Park 2016 |  |  | 11 |  |  | 11 | 22 |  | |
| 7. Tan et al., (2022) | Park 2016a | 5.8 | 2.6 | 11 | 4.8 | 1.8 | 5 | 16 |  | |
| 9. Xie et al., (2022) | Park 2016a | 70.15 | 16.04 | 11 | 63.43 | 15.3 | 5 | 16 |  | |
| 15. Bath et al. (2018) | Park 2016a |  |  | 11 |  |  | 5 | 16 |  | |
| 7. Tan et al., (2022) | Park 2016b | 3.8 | 2.7 | 11 | 4.8 | 1.8 | 6 | 17 |  | |
| 9. Xie et al., (2022) | Park 2016b | 44.8 | 22.02 | 11 | 63.43 | 15.3 | 5 | 16 |  | |
| 15. Bath et al. (2018) | Park 2016b |  |  | 11 |  |  | 6 | 17 |  | |
| 1. Balcerak et al. (2022) | Park 2017 |  |  |  |  |  |  | 35 |  | |
| 5. Li et al., (2022) | Park 2017 | 25.3 | 17.95 | 22 | 7 | 14.66 | 11 | 33 |  | |
| 6. Qiao et al., (2022) | Park 2017 | -2.24 | 2.1 | 22 | -1.2 | 0.85 | 11 | 33 |  | |
| 13. Yang et al. (2021) | Park 2017 |  |  | 11 |  |  | 11 | 22 |  | |
| 13. Yang et al. (2021) | Park 2017 |  |  | 11 |  |  | 11 | 22 |  | |
| 16. Chiang et al. (2018) | Park 2017 |  |  | Unclear |  |  | Unclear | Unclear |  | |
| 4. Hsiao et al., (2023) | Park 2017 | -0.29 | 0.71 | 11 | -0.71 | 0.71 | 11 | 22 |  | |
| 12. Wang et al. (2021) | Park 2017a |  |  | 9 |  |  | 9 | 18 |  | |
| 14. Cheng et al. (2021) | Park 2017a |  |  | 11 |  |  | 11 | 22 |  | |
| 12. Wang et al. (2021) | Park 2017b |  |  | 11 |  |  | 12 | 23 |  | |
| 14. Cheng et al. (2021) | Park 2017b |  |  | 11 |  |  | 11 | 22 |  | |
| 3. Banda et al., (2023) | Tageldin 2020 |  |  |  |  |  |  | 18 |  | |
| 3. Banda et al., (2023) | Tarameshlu 2014 |  |  |  |  |  |  | 18 |  | |
| 1. Balcerak et al. (2022) | Tarameshlu 2019 |  |  |  |  |  |  | 18 |  | |
| 5. Li et al., (2022) | Tarameshlu 2019 | 1 | 0.74 | 6 | 0 | 0.85 | 6 | 12 |  | |
| 6. Qiao et al., (2022) | Tarameshlu 2019 | -0.9 | 0.8 | 6 | 0 | 0.92 | 6 | 12 |  | |
| 9. Xie et al., (2022) | Tarameshlu 2019 | 2 | 0.478 | 6 | 2.52 | 1.91 | 6 | 12 |  | |
| 14. Cheng et al. (2021) | Tarameshlu 2019a |  |  | 6 |  |  | 6 | 12 |  | |
| 14. Cheng et al. (2021) | Tarameshlu 2019b |  |  | 6 |  |  | 6 | 12 |  | |
| 7. Tan et al., (2022) | Terameshlu 2018a | 3.75 | 1.67 | 6 | 4.25 | 1.67 | 3 | 9 |  | |
| 7. Tan et al., (2022) | Terameshlu 2018b | 2.5 | 0.74 | 6 | 4.25 | 1.67 | 3 | 9 |  | |
| 3. Banda et al., (2023) | Unluer 2019 |  |  | 15 |  |  | 13 | 28 |  | |
| 4. Hsiao et al., (2023) | Unluer 2019 | -2.87 | 1.63 | 15 | -2.38 | 1.59 | 13 | 28 |  | |
| 5. Li et al., (2022) | Unluer 2019 | 2.87 | 2.53 | 15 | 2.38 | 2.51 | 13 | 28 |  | |
| 6. Qiao et al., (2022) | Unluer 2019 | -2.87 | 2.53 | 15 | -2.38 | 2.51 | 13 | 28 |  | |
| 7. Tan et al., (2022) | Unluer 2019 | 9.11 | 9 | 9 | 12.6 | 7.91 | 7 | 16 |  | |
| 11. Li et al. (2021) | Unluer 2019 |  |  | 15 |  |  | 13 | 28 |  | |
| 12. Wang et al. (2021) | Unluer 2019 |  |  | 15 |  |  | 13 | 28 |  | |
| 13. Yang et al. (2021) | Unluer 2019 |  |  | 15 |  |  | 13 | 28 |  | |
| 14. Cheng et al. (2021) | Unluer 2019 |  |  | 15 |  |  | 13 | 28 |  | |
| 4. Hsiao et al., (2023) | Zhang 2019 | -10 | 11.43 | 16 | -5.24 | 7.61 | 16 | 32 |  | |
| 5. Li et al., (2022) | Zhang 2019 | 11.02 | 10.82 | 48 | 4.94 | 7.47 | 16 | 64 |  | |
| 4. Hsiao et al., (2023) | Zhang 2019 | -9.52 | 10 | 16 | -5.24 | 7.62 | 16 | 32 |  | |
| 14. Cheng et al. (2021) | Zhang 2019a |  |  | 16 |  |  | 16 | 32 |  | |
| 14. Cheng et al. (2021) | Zhang 2019b |  |  | 16 |  |  | 16 | 32 |  | |
| 14. Cheng et al. (2021) | Zhang 2019c |  |  | 16 |  |  | 16 | 32 |  | |
| 11. Li et al. (2021) | Zhang 2020 |  |  | 14 |  |  | 15 | 29 |  | |
| 8. Wen et al., (2022) | Zhang 2020a | 2.23 | 0.51 | 13 | 1 | 0.45 | 7 | 20 |  | |
| 8. Wen et al., (2022) | Zhang 2020b | 2.5 | 0.53 | 14 | 1 | 0.45 | 8 | 22 |  | |
| 11. Li et al. (2021) | Zheng 2017 |  |  | 45 |  |  | 45 | 90 |  | |
| 3. Banda et al., (2023) | Zhong 2021 |  |  | 36 |  |  | 35 | 71 |  | |
| 5. Li et al., (2022) | Zhong 2021 | 0.63 | 1.1 | 74 | 0.29 | 0.79 | 35 | 109 |  | |
| 6. Qiao et al., (2022) | Zhong 2021 | -1.37 | 1.9 | 108 | -0.23 | 1.39 | 35 | 143 |  | |
| 11. Li et al. (2021) | Zhong 2021 |  |  | 38 |  |  | 35 | 73 |  | |
| 8. Wen et al., (2022) | Zhong 2021a | 1.44 | 0.4 | 38 | 0.23 | 0.33 | 18 | 56 |  | |
| 8. Wen et al., (2022) | Zhong 2021b | 1.16 | 0.47 | 36 | 0.23 | 0.33 | 17 | 53 |  | |
| 2. Papadopoulou et al. (2018) |  |  |  |  |  |  |  |  |  | |
| Key: CTR_m: Control Mean Scores; CTR_n: Control Total Number of Participants; CTR_sd: Control Standard Deviation; TMS_m: Transcranial Magnetic Stimulation Mean Scores; TMS_n: Transcranial Magnetic Stimulation Total Number of Participants; TMS_sd: Transcranial Magnetic Stimulation Standard Deviation; Total_n: Total Number of Participants | | | | | | | | | |  |

**Supplementary Material 5**

Statistics variation in primary studies across multiple meta-analyses
